# Influence of Degree of Severe Plastic Deformation on Thermal Stability of an HfNbTiZr Multi-Principal Element Alloy Processed by High-Pressure Torsion

**DOI:** 10.3390/nano12193371

**Published:** 2022-09-27

**Authors:** Pham Tran Hung, Megumi Kawasaki, Ábel Szabó, János L. Lábár, Zoltán Hegedűs, Jenő Gubicza

**Affiliations:** 1 Department of Materials Physics, Eötvös Loránd University, P.O.B. 32, 1518 Budapest, Hungary; 2 School of Mechanical, Industrial and Manufacturing Engineering, Oregon State University, Corvallis, OR 97331, USA; 3 Institute for Technical Physics and Materials Science, Centre for Energy Research, 1121 Budapest, Hungary; 4 Deutsche Elektronen-Synchrotron DESY, 22607 Hamburg, Germany

**Keywords:** HfNbTiZr multi-principal element alloy, severe plastic deformation, annealing, dislocations, hardness

## Abstract

Severe plastic deformation (SPD) is an effective route for the nanocrystallization of multi-principal element alloys (MPEAs). The stability of the refined microstructure is important, considering the high temperature applications of these materials. In the present study, the effect of SPD on the stability of a body-centered cubic (bcc) HfNbTiZr MPEA was investigated. SPD was performed using a high-pressure torsion (HPT) technique by varying the number of turns between ½ and 10. The evolution of phase composition and microstructure was studied near the disk centers and edges where the imposed strain values were the lowest and highest, respectively. Thus, the shear strain caused by HPT varies between 3 (½ turn, near the center) and 340 (10 turns, near the edge). It was found that during annealing up to 1000 K, the bcc HfNbTiZr alloy decomposed into two bcc phases with different lattice constants at 740 K. In addition, at high strains a hexagonal close packed (hcp) phase was formed above 890 K. An inhomogeneous elemental distribution was developed at temperatures higher than 890 K due to the phase decomposition. The scale of the chemical heterogeneities decreased from about 10 µm to 30 nm where the shear strain increased from 3 to 340, which is similar to the magnitude of grain refinement. Anneal-induced hardening was observed in the MPEA after HPT for both low and high strains at 740 K, i.e., the hardness of the HPT-processed samples increased due to heat treatment. At low strain, the hardness remained practically unchanged between 740 and 1000 K, while for the alloy receiving high strains there was a softening in this temperature range.

## 1. Introduction

Multi-principal element alloys (MPEAs), which include high-entropy alloys (HEAs), are materials that comprise several constituents with similar fractions [1]. These emerging materials are rapidly gaining attention from the science community [2], partly due to the realization of the vast number of new alloys, but also owing to the superior properties possessed by these novel materials. It has been reported that MPEAs exhibit excellent mechanical properties, such as high hardness [3,4,5], good fatigue, wear and corrosion resistance [6,7,8], etc. Among them, refractory MPEAs are typically well-suited for high temperature applications, thanks to the high melting points of the refractory elements (above 1875 °C). While many refractory MPEAs display high strength even at high temperatures, typically they are also brittle at room temperature [9], limiting the range of application. An equimolar refractory HfNbTiZr MPEA was reported to demonstrate not only good hardness even at high temperatures, but also good ductility at ambient temperatures [10].

Other than selecting the chemical composition of the material, tailoring the microstructure is a well-known approach for obtaining the desired mechanical properties. MPEAs are usually produced via a melting method, yielding coarse-grained materials [11,12,13,14,15]. The strength of such materials can be enhanced by severe plastic deformation (SPD), which increases the amount of defects (e.g., dislocations) and decreases grain size. Among the SPD methods, high-pressure torsion (HPT) is capable of producing the highest imposed strain [5,16,17,18]. In particular, applying 10 HPT turns to an HfNbTiZr MPEA has been shown to refine the microstructure from a coarse-grained state to the nanocrystalline regime, while the hardness increased by 70% from 2630 MPa to 4450 MPa [19].

Investigating the thermal responses of MPEAs is not only beneficial when designing suitable heat-treatment processes for tailoring the microstructure, but also critical for high-temperature applications. The thermal stability of MPEAs depends on both the chemical composition and the microstructure. For example, an as-cast single phase HfNbTiZr MPEA was stable at 673 K for 100 h, while precipitates appeared after 200 h at the same temperature, and after 700 h the material consisted of two bcc phases and an additional hcp phase [20]. Cao et al. [21] performed annealing for 100 h at different temperatures on the same MPEA, confirming that at 673 K elemental segregation occurs, but there was no formation of extra crystalline phases. On the other hand, precipitations were observed by electron microscopy at 773, 873 and 1023 K. At 1173 K, the MPEA became single phase again without elemental partition [21]. By contrast, in an HfNbTiZr MPEA cold-rolled to a cross-sectional area reduction of 80%, bcc and hcp precipitates were formed at 723 K even after 10 h of aging; however, if the rolled microstructure was intentionally recrystallized, precipitation occurred only after 200 h at 723 K [22]. In our prior paper [5], it was also demonstrated that a nanocrystalline HfNbTiZr processed by HPT for 10 turns decomposed into two bcc phases when annealed up to 740 K at a heating rate of 40 K/min, and an additional hcp phase appeared at 890 K.

**Figure 1:**
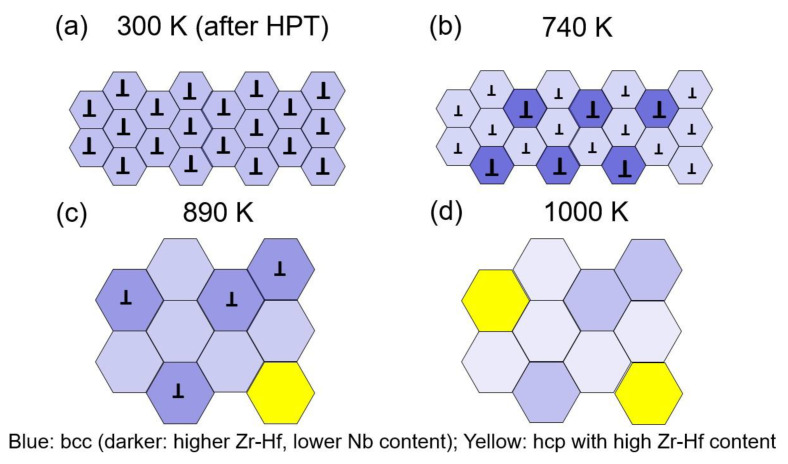
Schematic showing the evolution of microstructure at the edge of a nanocrystalline HfNbTiZr MPEA disk processed by 10 HPT turns (**a**) followed by heating up to the temperatures of 740 (**b**), 890 (**c**) and 1000 K (**d**), which is adapted from the experimental results presented in [5]. The blue and yellow hexagons indicate grains with bcc and hcp structures. The inverted “T” represents dislocations inside the grains. The larger the size of inverted “T”, the higher the dislocation density.

In this paper, the effect of the strain imposed during HPT on the thermal stability of an HfNbTiZr MPEA is investigated. This work is an extension of our former study [5], where microstructure and phase-composition changes were investigated at the edge of a nanocrystalline HfNbTiZr MPEA disk processed for 10 HPT turns followed by heating up to temperatures of 740, 890 and 1000 K. These temperatures were selected on the basis of a differential scanning calorimetry (DSC) measurement. It was found that at 740 K two bcc phases were formed in the sample, while at 890 K an additional hcp phase also appeared. Moreover, a recovery occurred in the bcc phases during annealing. At 1000 K, there was further recovery and recrystallization, as well as an increase in the hcp phase fraction. The microstructure evolution during annealing is shown schematically in Figure 1. In the current work, our former study focusing on one position per HPT disk is extended to two locations of the disk center and edge as well as to other HPT turns, namely ½, 1, 5 and 10 HPT turns. Due to the extension, the measurement locations in the alloy involve shear strain in a wide range, between ~3 and ~340. Our goal is to reveal the effect of SPD on the thermal stability of HfNbTiZr MPEA by systematically evaluating the microstructural changes at the locations with different shear strains. For the sample preparation, all processed samples are annealed at the same temperatures applied in our previous work [5]. Investigation of the phase composition and microstructure of the annealed specimens was conducted, and the relation with the measured hardness is discussed in this report.

## 2. Materials and Methods

### 2.1. Processing of MPEA and Annealing Conditions

A refractory Hf_25_Nb_25_Ti_25_Zr_25_ MPEA was synthesized via magnetic levitation melting, using a mixture of four pure components (purity > 99.9 wt%). The process is followed by a heat treatment at 1290 °C for 24 h in order to homogenize the material. Afterward, cylindrical billets with a diameter of 10 mm were machined from the cast material, which were then subsequently sliced into 1-mm-thick disks using electric discharge machining (EDM). A series of mechanical grindings was applied to achieve disks with a final thickness of ~0.85 mm. The HPT process was performed using a conventional HPT facility with quasi-constrained set-up [23]. The numbers of HPT turns were ½, 1, 5 and 10, conducted at a rotational speed of 1 rpm under a pressure of 6.0 GPa at room temperature.

In order to study the annealing effect on the material, a Perkin Elmer (DSC2) calorimeter (manufacturer: Perkin Elmer, Waltham, MA, USA) was employed to perform the heat-treatment process. The targeted temperatures were selected as in our prior paper [5], namely 740, 890 and 1000 K. Individual specimens were heated up to the chosen temperatures with a 40 K/min heating rate and immediately quenched with a 300 K/min quenching rate to room temperature for microstructure investigation.

### 2.2. Study of Phase Composition by X-ray Diffraction

The microstructure and phase composition were investigated by X-ray diffraction (XRD) at two different distances from the disc center of each HPT-processed sample. Small pieces near the center and edge of the disks were cut using a diamond saw. Due to the need to prepare multiple samples from each disk to anneal at different temperatures, it was not possible to have a specimen at exactly the center of the HPT disks. Therefore, the middle of the “center” and “edge” samples were located at ~0.75 mm and ~4.35 mm from the actual disk center, respectively. The widths of the specimens were ~1.1 mm and ~1.3 mm for the “center” and “edge” samples, respectively, and both of them had a height of ~3 mm (the dimension of the cut-pieces perpendicular to the radial direction of the HPT-processed disks). Even though these locations are about 0.65–0.75 mm away from the true center and edge of the HPT disks, these specimens will be referred to as “center” and “edge” samples in this paper.

Before the XRD measurements, the surfaces of the specimens were treated with mechanical and electropolishing. The mechanical polish was performed using a series of SiC papers with a grit of 800, 1200, 2400 and 4000, and afterward with a colloidal alumina suspension having 1 μm particle size. The samples are then etched using a solution of 45 mL distilled water, 5 mL HF and 2 mL nitric acid for ~30 s to remove the outmost surface layer distorted by mechanical polishing.

A Smartlab powder diffractometer (manufacturer: Rigaku, Tokyo, Japan) with CuKα radiation having a wavelength of λ = 0.15418 nm was utilized for the investigation of the phase composition of the HPT-processed and the annealed HfNbTiZr MPEA samples. The diffractometer was used in Bragg–Brentano geometry. In all annealed samples, two bcc phases with overlapping peaks were observed, and some samples had an additional hcp phase. The overlapping peaks of the different phases were separated by their fitting with a sum of Lorentzian functions. The fraction of each phase was estimated as the fraction of the sum of the intensity of the corresponding peaks over all measurable peaks on the XRD pattern. The lattice constant was calculated from the diffraction peak position by utilizing the Nelson–Riley method [24].

### 2.3. Investigation of the Microstructure by X-ray Line Profile Analysis

**Figure 2:**
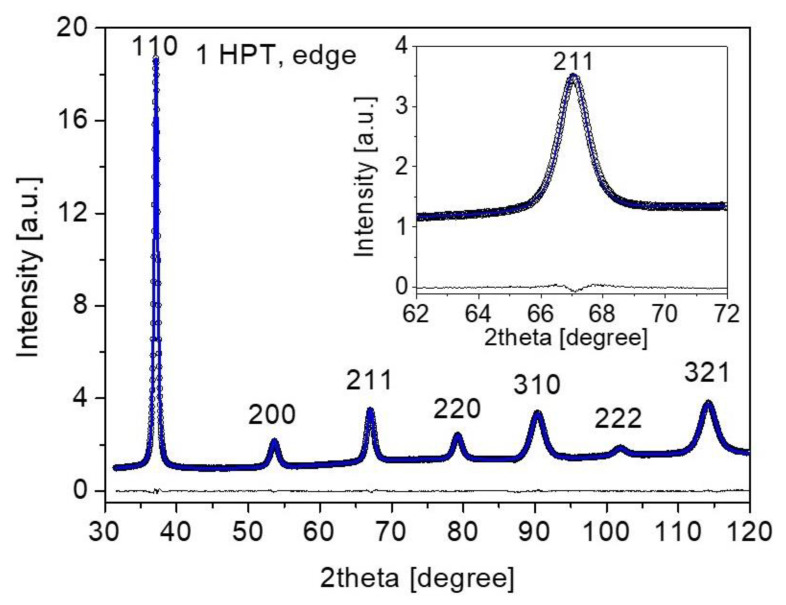
CMWP fitting on the XRD pattern taken at the edge part of the sample processed by 1 turn of HPT without any annealing. The open circles and the blue line correspond to the measured and fitted diffractograms, respectively, while the difference between them is indicated as a line at the bottom of each plot. The indices of reflections are also shown. The inset shows reflection 211 with a higher magnification.

X-ray line-profile analysis (XLPA) was applied for the characterization of the microstructure of the MPEA samples. A diffractometer with a rotating anode (type: RA-MultiMax9, manufacturer: Rigaku, Tokyo, Japan) with two-dimensional imaging-plate detectors was employed for the XLPA measurement, using a CuKα_1_ radiation with a wavelength of λ = 0.15406 nm. The convolutional multiple whole profile (CMWP) fitting method [25] was used to analyze the line profiles. This procedure considers the measured diffraction pattern as a sum of a background spline and the theoretical peaks obtained as the convolution of the profiles associated with the diffraction domain size and the dislocation density. The fitting was performed using a gradient descend method to minimize the sum of the squared residuals between the measured and theoretical patterns. Due to the presence of dislocations in the SPD-deformed microstructure, the diffraction peaks exhibited strain anisotropy, i.e., their breadth depended significantly on the indices of reflections, which can be expressed with the dislocation contrast factors [25]. The contrast factors for the bcc HfNbTiZr MPEA depend on two parameters, namely *C*_h00_, which has a value of ~0.31 for both edge and screw dislocations, and *q*, which has values of 1.5 and 2.7 for edge and screw dislocations, respectively [19]. The hcp phase of the MPEA was not evaluated with XLPA due to its low intensity. Figure 2 demonstrates a fitting using the CMWP method on the edge part of the sample processed by 1 turn of HPT without any annealing.

The in-depth homogeneity of the microstructure of the MPEA after HPT was checked by XLPA using synchrotron X-ray diffraction, carried out at the Swedish Materials Science beam line (P21.2) at the Deutsches Elektronen Synchrotron (DESY). A beam focused to a size of 2.2 × 7 μm^2^ with a beam energy of 82.5 keV (wavelength: λ = 0.01503 nm) was utilized. Disk centers and edges after HPT for ½ and 10 turns were measured through the full thickness of the HPT disks with a step size of 10 μm. During the collection of each diffraction profile, the sample was moved perpendicularly to the beam and parallel to the disk diameter by ±100 μm to increase the scattering volume and obtain better statistics. The calibration of the distance between the sample and detector (995 mm) was carried out with a standard LaB_6_ powder (NIST SRM660c) and the recording of the patterns was conducted with a flat panel detector with 430 × 430 mm^2^ active area and 150 × 150 μm^2^ pixel size (type: Varex XRD 4343CT, manufacturer: Varex Imaging, Salt Lake City, UT, USA). The 2-dimensional intensity distribution recorded by the detector were integrated along the Debye–Scherrer rings to obtain the usual (intensity vs. diffraction angle) pattern using pyFAI software package (version 0.18, ESRF, Grenoble, France) [26].

### 2.4. Microstructure Study by Electron Microscopy

In order to investigate the influence of plastic deformation on the thermal responses of the HfNbTiZr MPEA, an examination of the microstructure with electron microscopy was conducted for the specimens with the lowest and highest imposed strains, i.e., for the center of the disk processed by ½ HPT turn and the edge of the sample deformed by 10 HPT turns. For the center part of the disk after a half HPT turn and its annealed counterparts, the microstructure characterization was carried out via electron backscatter diffraction (EBSD). For this inspection, an FEI Quanta 3D scanning electron microscope (SEM) was utilized (manufacturer: Thermo Fisher Scientific, Waltham, MA, USA). In order to prepare the surface for the EBSD measurement, a SEMPrep (SC-1000) device from Technoorg Linda (Budapest, Hungary) was used for conducting ion milling for ~30 min, applying an Ar ion beam and starting with an acceleration voltage of 10 keV which was then reduced to 1 keV. The EBSD images are taken using step sizes between 0.5 and 2 μm depending on the fineness of the microstructure, and were analyzed using Orientation Imaging Microscopy (OIM) software (manufacturer: TexSem Laboratories, Provo, UT, USA). For the examination of the spatial elemental distribution, energy-dispersive X-ray spectroscopy (EDS) measurement was conducted using the same SEM facility.

For the specimen taken from the edge of the disk processed by 10 HPT turns leading to true nano-scale grain size and high defect density, transmission electron microscopy (TEM) and EDS were exploited for the microstructure characterization of both the HPT-processed and annealed samples. In order to prepare thin TEM-lamellae from the bulk samples, the focused ion beam (FIB) technique was used with the help of Ga^+^ ions. The specimens were first mounted onto a Cu stub for mechanical grinding and polishing using a special glue at 100 °C for not more than 1 min. Afterward, an ion beam with an acceleration voltage of 7 keV and a current of 2 nA was employed for thinning the lamellae with a starting thickness of 50 μm, and then a reduced voltage of 3 keV, and lastly 1 keV were applied on both sides of the lamellae to eliminate the damages on the surfaces. After the lamella preparation, the TEM investigation was carried out using a Titan Themis G2 200 scanning transmission electron microscope (STEM), equipped with a four-segment Super-X EDS detector (manufacturer: Thermo Fisher Scientific, Waltham, MA, USA). The imaging part was corrected for the spherical aberration (C_s_), while no probe-correction was applied. The images were recorded with a Fishione high-angle annular dark-field (HAADF) detector (manufacturer: Thermo Fisher Scientific, Waltham, MA, USA), with the image resolution of 0.16 nm in STEM Z-contrast imaging mode, and with a 4 k × 4 k CETA 16 M indirect detection CMOS camera (Thermo Fisher Scientific, Waltham, MA, USA) in both bright-field (BF) and dark-field (DF) modes. The EDS data of the studied MPEA was recorded in spectrum-image mode (together with the HAADF signal). The average grain size was determined by counting about 40–180 grains for each sample using the DF images, since these are more suitable for the identification of the individual grains compared to the BF micrographs. In practice, the numbers of the measured grains were between 34 and 183 for the different HPT-processed and annealed samples.

### 2.5. Hardness Test

A surface treatment was conducted before the hardness test, similar to the process performed before the XRD measurement (see Section 2.2). The indentation was performed using a Zwick Roell ZHμ hardness tester with a Vickers indenter (manufacturer: Kennesaw, GA, USA). The load and the dwell time were 500 g and 10 s, respectively, for all samples after HPT and annealing. For each sample, 10 measurements were carried out, and the average was used as the final microhardness value.

## 3. Results

### 3.1. Microstructure Evolution with Increasing Straining during HPT

**Figure 3:**
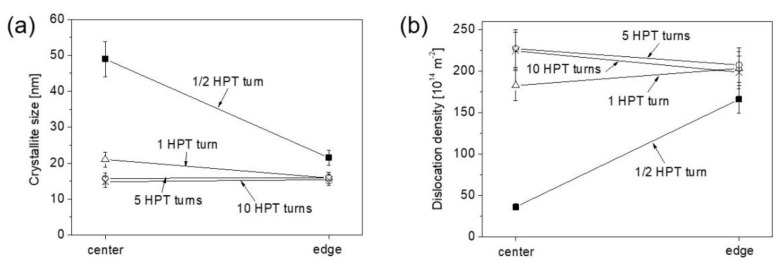
The crystallite size (**a**) and the dislocation density (**b**) measured by XLPA at the centers and edges of the disks after ½, 1, 5 and 10 HPT turns.

**Figure 4:**
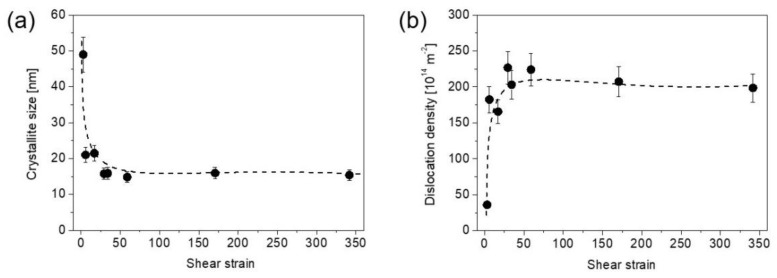
The crystallite size (**a**) and the dislocation density (**b**) measured by XLPA vs. the shear strain evolved during HPT. The dashed curves are just a guide for the eyes.

Figure 3a,b show the crystallite size and the dislocation density measured by XLPA at both center and edge of the samples after ½, 1, 5 and 10 HPT turns. For a half turn, the crystallite size at the center is about 50 nm which decreased to ~20 nm at the edge. When the number of turns increased to one, the crystallite size decreased to ~20 and ~15 nm at the disk center and edge, respectively. For 5 and 10 turns, the crystallite sizes were about 15 nm for both locations at the center and periphery. Regarding the dislocation density, it increased from ~36 × 10^14^ m^−2^ to ~166 × 10^14^ m^−2^ from the disk center to the edge, respectively, after a half turn. The dislocation density increased to ~182 × 10^14^ m^−2^ at the disk center after one turn, while at the edge it reached a value of about 200 × 10^14^ m^−2^. After 5 and 10 turns, the dislocation density saturated at ~220 × 10^14^ m^−2^ in both the center and edge parts of the disks. Figure 4a,b show the crystallite size and the dislocation density vs. the shear strain (γ) evolved during HPT, which can be determined as:

(1) $$\gamma =\frac{2\pi rn}{t},$$

where *n* is the number of turns, *r* is the distance from the disk center and *t* is the thickness of the specimen [27]. This study uses *t* ≈ 0.8 mm for all numbers of turns, and *r* = 0.75 and 4.35 for the center and edge positions, respectively, examined for the disks. Based on Equation (1), the lowest and the highest shear-strain values in the present study were ~3 and ~340, respectively, which are characteristic at the disk center for ½ turn and edge for 10 turns. Figure 4 reveals that the crystallite size decreased while the dislocation density increased with increasing the shear strain and both quantities saturated at the strain of about 30 with the values of about 15 nm and ~220 × 10^14^ m^−2^, respectively. It should be noted that the crystallite (or coherently scattering domain) size obtained by XLPA is smaller than the grain size determined by either TEM or EBSD, as will be shown in the next section. This phenomenon is usual for SPD-processed metallic materials and caused by the fact that even very small misorientations can break the coherency of X-rays scattered from the material [28]. Therefore, for hierarchical refined microstructures obtained by SPD, the crystallite size determined by XLPA corresponds to the dimension of dislocation cells or subgrains rather than the grain size [29,30].

**Figure 5:**
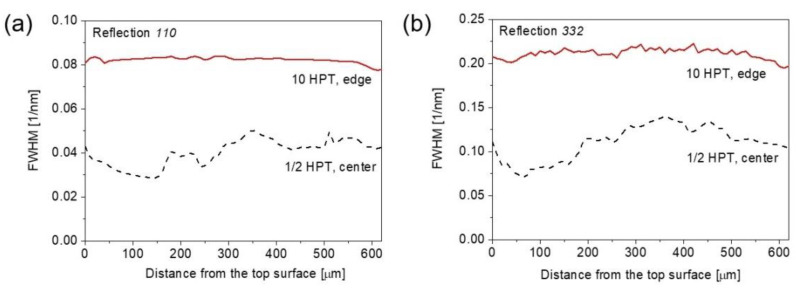
The full width at half maximum (FWHM) of 110 (**a**) and 332 (**b**) diffraction peaks as a function of the distance from the top surface of the disks processed for ½ and 10 turns.

It is worth noting that laboratory XRD investigated only an about 10-µm-thick surface layer of the HPT-processed disks. The question arises whether the microstructural parameters measured on the surface describe the whole disk well, since there may be heterogeneous structural changes along the through-thickness direction during HPT. To clarify this question for the bcc MPEA, a through-thickness characterization of the microstructure was carried out by synchrotron XRD. Namely, 1-mm-thick lamellas were cut parallel to the disk diameter and XRD patterns were measured in transmission diffraction configuration on the cross-section of the disks as a function of the distance from the top surface. These experiments were performed on the samples involving the lowest and highest strains, i.e., at the center of the disk processed for ½ turn and the edge of the sample after 10 turns. As an example, Figure 5a,b show the full width at half maximum (FWHM) of 110 and 332 diffraction peaks, respectively, as a function of the distance from the top surface of the disks. Reflections 110 and 332 are the 1st and the 11th peaks of the bcc phase, respectively, i.e., they were taken from the beginning and at the end of the diffraction patterns. There was no strong variation in the peak breadth vs. the distance from the top surfaces, especially for the edge after 10 turns. Thus, it can be concluded that the microstructure was reasonably homogeneous along the thickness of the HPT disks for both the lowest and highest strains, implying that the quantitative results (i.e., the crystallite size and the dislocation density) obtained on a surface layer by laboratory XRD also characterize the material inside the disks.

### 3.2. Effect of Heat Treatment on the Phase Composition Revealed by XRD

**Figure 6:**
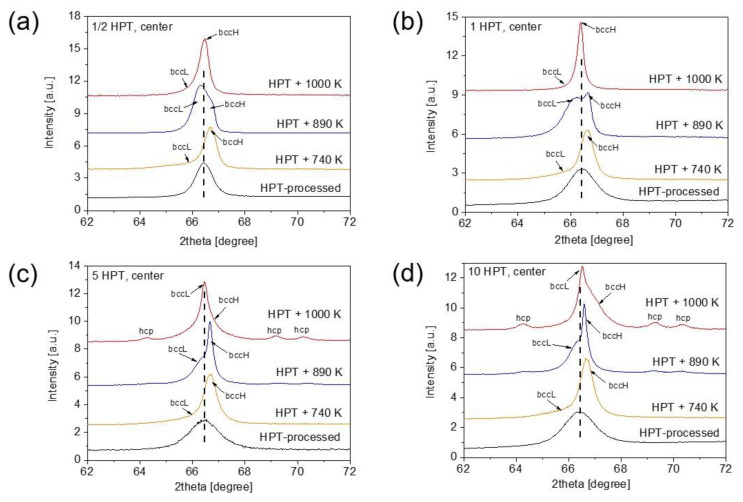
Evolution of a part of the XRD pattern between the diffraction angles of 62 and 72° for the center parts of the disks processed for (**a**) ½, (**b**) 1, (**c**) 5 and (**d**) 10 HPT turns and annealed at various temperatures.

**Figure 7:**
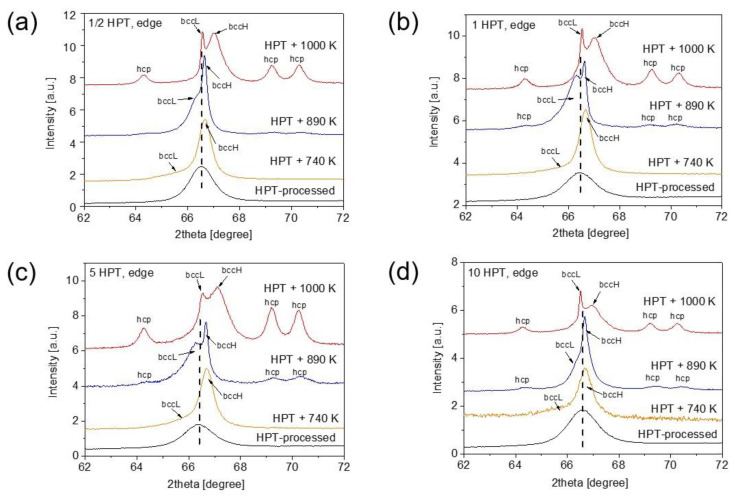
Evolution of a part of the XRD pattern between the diffraction angles of 62 and 72° for the edge parts of the disks processed for (**a**) ½, (**b**) 1, (**c**) 5 and (**d**) 10 HPT turns and annealed at various temperatures.

Figure 6 shows the evolution of a part of the XRD pattern between the diffraction angles of 62 and 72° for the disk center processed for different HPT turns and annealed at various temperatures. For the lowest number of turns (1/2), the bcc peak with the indices 211 was separated to two profiles at 740 K. This peak splitting was most probably caused by the development of chemical heterogeneities, i.e., the decomposition of the single bcc phase into two bcc structures with different lattice constants. The bcc phases related to the diffraction peaks at lower and higher angles are denoted as bccL and bccH, respectively. The edge parts of the disks also demonstrated the decomposition of the bcc phase to bccL and bccH phases at 740 K as shown in Figure 7. It is noted that the peak splitting was observed for all XRD reflections. The lattice constant of the bccL phase is higher, while that for the bccH phase is lower, than the average lattice parameter of the HPT-processed, more homogeneous structure. For instance, in the case of the edge of the disk processed for 10 turns and then annealed to 740 K, the lattice constants of the bccL and bccH phases are 0.3485 ± 0.0007 and 0.3427 ± 0.0001 nm, respectively, in comparison with the value of 0.3438 ± 0.0003 nm determined immediately after HPT. The different lattice parameters of the bccL and bccH phases could have resulted in the difference in their chemical compositions. Indeed, Hf and Zr have similar and high atomic radii (about 155 pm) compared to the other two elements of the studied alloy. In fact, the radii of Nb and Ti are 7% and 11% smaller than those for Hf or Zr. Therefore, the XRD peak positions suggest that the bccL phase is enriched in Hf and/or Zr while the bccH phase has an elevated concentration of Nb and/or Ti. The EDS results on the chemical composition will be presented later.

**Figure 8:**
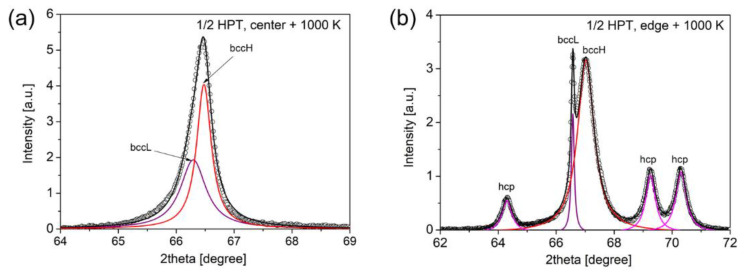
Illustration of the separation of the overlapping XRD peaks of bccL and bccH phases by fitting a measured profile with the sum of two Lorentzian functions (**a**). This method was used in the determination of the phase fractions from the peak areas. When three phases (bccL, bccH and hcp) coexist, the separation of their peaks using the Lorentzian fitting is illustrated in (**b**).

The fractions of the bccL and bccH phases were determined from the integrated intensities of their diffraction peaks in the angle range between 30 and 150°. As an example, Figure 8a illustrates the separation of the peaks of bccL and bccH phases by fitting a measured profile with the sum of two Lorentzian functions for the disk center after HPT for a half turn followed by annealing up to 1000 K. This fitting was performed for all experimental peaks in the patterns. Then, the areas under the peaks for each phase were summed up and the phase fractions were determined from these integrated intensities. At 740 K, as shown in Figure 6 and Figure 7, bccH is the major phase with a fraction of 70–80% for both the center and edge of the disks, irrespectively of the number of turns.

With increasing temperature from 740 K to 890 K, the peak position of the bccH phase only marginally changed. On the other hand, the peaks of the bccL phase were shifted to higher angles, indicating the decrease in lattice constant compared to the value measured at 740 K. These observations are valid for both the center and edge parts of the disks for all numbers of turns. For instance, at the edge of the disk processed for 10 turns, a large decrease in the lattice parameter of the bccL phase is observed from 0.3485 ± 0.0007 to 0.3443 ± 0.0002 nm, while the lattice constant of the bccH phase only slightly changed from 0.3427 ± 0.0001 to 0.3435 ± 0.0001 nm, with a temperature change from 740 to 890 K.

**Figure 9:**
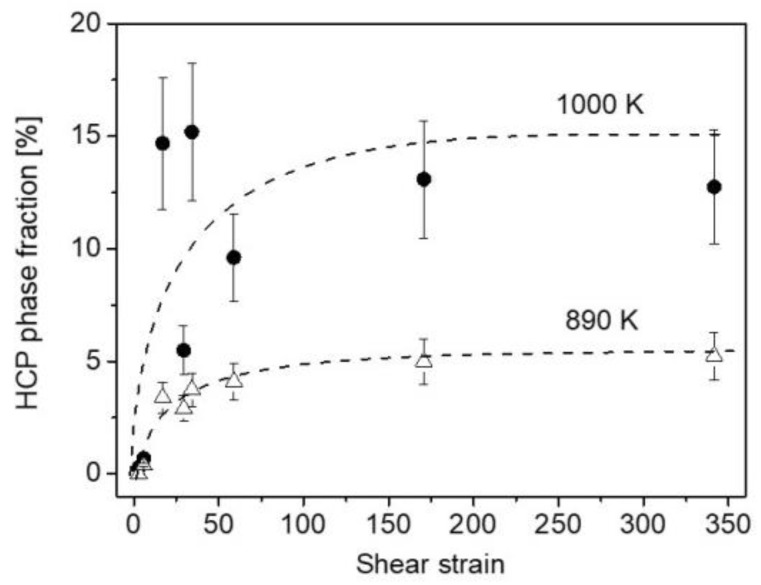
Evolution of the hcp phase fraction vs. the shear strain due to annealing at 890 (open triangles) and 1000 K (solid circles). The dashed curves are just a guide for the eyes.

Contrary to the lattice parameters, the phase fractions at 890 K depend strongly on the degree of SPD straining. Namely, for low strains (e.g., at the disk center after a half HPT turn) the bccL phase became the main phase with a fraction of about 80% (see Figure 6a). With increasing numbers of turns at the center, the fraction of the bccL phase decreased and an additional hcp phase was formed as shown in Figure 6. Figure 7 reveals that at the disk edges, the peaks of the hcp phase appeared in the alloy processed for all numbers of turns. The fraction of the bccL phase decreased to about 40% for the sample having the highest strain, i.e., at the edge of the disk processed for 10 turns. When the three phases (bccL, bccH and hcp) coexist, the separation of their peaks using the Lorentzian fitting is illustrated in Figure 8b. The evolution of the hcp phase fraction vs. the shear strain is plotted for annealing at 890 and 1000 K in Figure 9. At 890 K, the fraction of the hcp phase increased monotonously up to a strain of ~20 and saturated to the fraction of ~5%.

At 1000 K, except for the centers of the disks processed for one-half and one turn, an hcp phase was detected at all studied locations (see Figure 6 and Figure 7). The hcp fraction after annealing at 1000 K vs. the shear strain is plotted in Figure 9. Although the relationship between the hcp fraction and the strain is scattered, it is evident that the highest hcp fraction was greater (~15%) than for 890 K (~5%). Regarding the two bcc phases, the fraction of bccH was higher (about 60–80%) than the bccL phase for both low and high strains. On the other hand, the position and shape of the whole measured peak profile consisting of the bccL and bccH reflections were very different for low and high strains (see Figure 6 and Figure 7). Indeed, for low strains (i.e., for the centers of the disks processed for one-half and one turn) the peak of the bccH phase dominates and the contribution of the bccL phase is marginal. The position of the bccH peak at 1000 K is close to that detected immediately after HPT processing. The peak profile is relatively broad, despite annealing at high temperatures. For high strains (e.g., in the edge part of the disk processed for 10 turns), the bcc peak profile consists of a narrow bccL peak and a very broad bccH reflection (see Figure 7d). The position of the peak of the narrow bccL phase is close to that of the reflection detected after HPT. On the other hand, the broad bccH peak has a higher diffraction angle than the HPT-processed sample. This observation suggests a relatively small lattice parameter of the bccH phase, which may be caused by an enrichment in small elements such as Nb or Ti. For instance, for the bccH phase at the edge of the disk processed for 10 HPT turns and then annealed at 1000 K, the lattice constant was 0.3416 ± 0.0003 nm.

### 3.3. Microstructure Evolution during Annealing Obtained by XLPA

**Figure 10:**
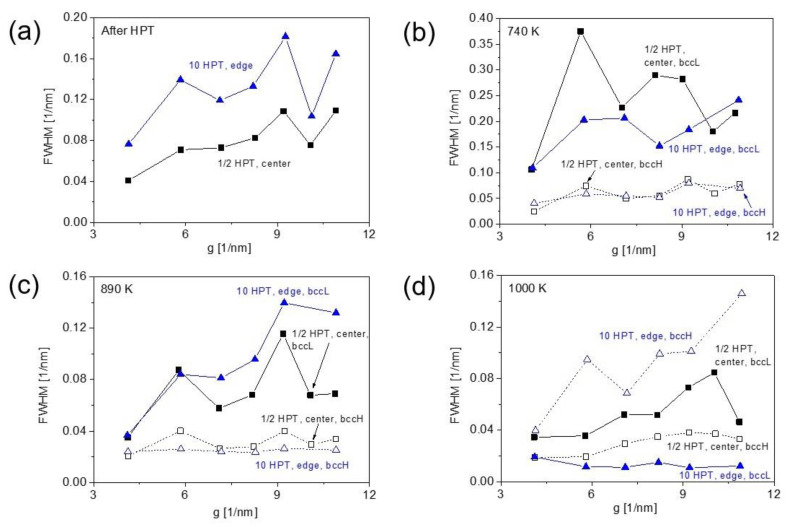
The full width at half maximum (FWHM) as a function of the length of the diffraction vector (g) for the bcc phases at the lowest and highest SPD strains applied in this study, i.e., for the disk centers and edges processed by HPT for ½ and 10 turns, respectively. (**a**) Immediately after HPT, (**b**) at 740 K, (**c**) at 890 K and (**d**) at 1000 K.

**Figure 11:**
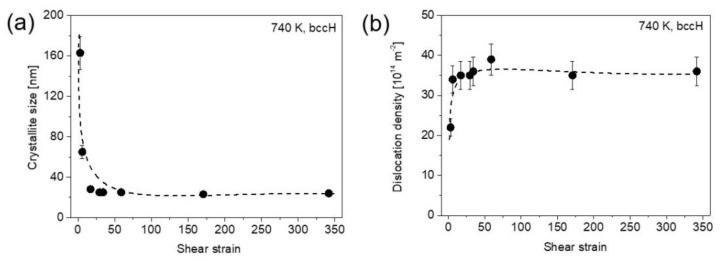
The crystallite size (**a**) and the dislocation density (**b**) vs. the shear strain for the bccH phase after annealing to 740 K. The dashed curves are just a guide for the eyes.

The XLPA evaluation of the XRD peaks can provide information about the change in the dislocation density and the crystallite size during heat treatment of the samples. On the other hand, a careful application of XLPA is suggested, since annealing may cause decomposition, and the chemical heterogeneities can also cause an XRD peak broadening, similar to dislocations [29]. Therefore, the density of dislocations and the crystallite size were determined by CMWP fitting only for those annealed bcc phases which do not show a significant increase in the peak breadth during the heat treatment. The increase in the dislocation density during the annealing of the SPD-processed metallic materials is not expected; therefore, peak broadening for the heat-treated alloy is most probably caused by chemical heterogeneities. Figure 10 shows the FWHM vs. the magnitude of the diffraction vector (g) for the materials with the lowest and highest SPD strains applied in this study, i.e., for the center and edge regions after ½ and 10 HPT turns, respectively. Comparing Figure 10a,b, it is evident for both samples with the lowest and highest applied strains that annealing at 740 K caused peak broadening for the bccL phase. Therefore, the patterns for this phase were not evaluated. On the other hand, the heat treatment at 740 K resulted in a significant decrease in the peak breadth for the bccH phase at the center and edge areas of the disks after ½ and 10 turns, respectively. Therefore, the patterns of this phase were evaluated by CMWP fitting and the results (crystallite size and dislocation density) vs. the shear strain are plotted in Figure 11. Comparing this figure with Figure 4, it is evident that the crystallite size is higher with a factor of two to three for the bccH phase after the heat treatment at 740 K than the condition immediately after HPT. In addition, the dislocation density is lower in bccH annealed at 740 K as compared to the HPT disks, especially for high strain values where the reduction is about sixfold from ~220 × 10^14^ m^−2^ to ~35 × 10^14^ m^−2^. These changes in the microstructure suggest recovery at 740 K. Similar to the HPT-processed state, the microstructural parameters of the crystallite size and the dislocation density in the bccH phase at 740 K saturated with the values of ~25 nm and ~35 × 10^14^ m^−2^, respectively, at the shear strain at ~20.

At 890 K, the bccH phase showed a reduced dislocation density with a value below ~3 × 10^14^ m^−2^, while the crystallite size values were between 30 and 60 nm. For the bccL phase at 890 K, the peak breadth at the center of the disk processed by a half turn was very similar to that obtained immediately after HPT (compare Figure 10a,c). Since recovery is expected at 890 K, which must yield a decrease in the peak breadth, the unchanged FWHM is most probably the result of a chemical inhomogeneity which enhanced peak broadening, thereby compensating the effect of recovery. Therefore, the microstructural evaluation by CMWP fitting of the peak profiles was not conducted for the bccL phase at 890 K.

After heating up to 1000 K, for the sample having low strains (e.g., in the center of the disk processed by a half turn) the main bccH phase exhibited considerable strain broadening of the XRD peak profiles as suggested by the WH plot in Figure 10d, since the peak breadth increased with the increasing length of the diffraction vector (g). The CMWP evaluation gave a significant value of dislocation density of ~5 × 10^14^ m^−2^ and the crystallite size was about 48 nm. On the other hand, for high strains, the peaks consisted of a narrow bccL and a very broad bccH profiles. The FWHM for bccH at 1000 K was similar to that observed for bccL at 890 K, as revealed by the comparison of Figure 10c,d. The peaks of the latter phase were not evaluated by XLPA, since most probably the chemical inhomogeneities caused a significant portion of broadening, as discussed in the previous paragraph; therefore, a similar effect can be expected for bccH at 1000 K, and thus the dislocation density was not determined from their peak profiles. On the other hand, the narrow XRD reflections of the bccL phase at 1000 K were evaluated by the CMWP method, and ~52 nm was obtained for the crystallite size, while the dislocation density was under the detection limit of XLPA (0.1 × 10^14^ m^−2^). This result is in accordance with the WH plot shown in Figure 10d, where strain broadening was not observed for the bccL phase at 1000 K. It can be concluded that recovery occurred only at the sample location involving low HPT strains followed by annealing at 1000 K, since the dislocation density was in the order of 10^14^ m^−2^, while at high strains at least a part of the material (namely the bccL phase) recrystallized.

### 3.4. Electron Microscopy Study for Examining the Heat-Treatment Effect on the Microstructure Processed by HPT for Low and High Strains

**Figure 12:**
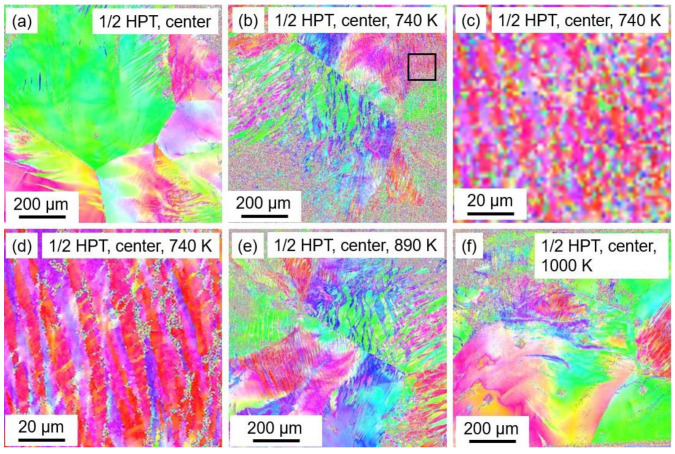
EBSD IPF maps obtained of the microstructure at the center of the disk processed by ½ turn (**a**) and subsequently annealed at 740 (**b**–**d**), 890 (**e**) and 1000 K (**f**). A part of the image in (**b**)—indicated by the black square—is magnified in (**c**) without changing the scan step size (2 µm). The same area is shown in (**d**) with a smaller step size of 0.5 µm.

For completing the XLPA study of the microstructure, electron microscopy investigations were performed on the alloys deformed by HPT for the lowest and highest applied strains (i.e., for the centers and the edges of the disk samples after ½ and 10 HPT turns, respectively). Figure 12 shows the EBSD IPF maps obtained of the microstructure at the center of the disk processed by a half turn and subsequently annealed at 740, 890 and 1000 K. In Figure 12a,b,e,f, the areas studied are 1 × 1 mm^2^ with a step size of 2 µm, and the left and right ends of the images correspond to the distances from the exact disk center of ~0.25 and ~1.25 mm, respectively. The investigation of such a large area was necessary for the lowest strain due to the relatively large initial grain size before HPT processing (~640 µm as shown in [19]). The boundaries of the initial large grains are still visible in the image taken immediately after one half turn of HPT (see Figure 12a). However, additional finer elongated grains were formed inside the initial grains. The grain size obtained from this EBSD image was 32 ± 10 µm. It is noticed that the grain size determined by EBSD is much larger than the crystallite size obtained by XLPA (about 50 nm) which is a usual tendency for SPD-processed metallic materials [28]. The explanation for this difference is given in Section 3.1. It is also noted that the error of the average grain sizes was determined as the ratio of the standard deviation and the square root of the number of studied grains.

Annealing at 740 K resulted in a reduction in grain size to 15 ± 2 µm. Indeed, the initial grains are more fragmented in Figure 12b than in Figure 12a. The reduced grain size during the heat treatment at 740 K will be discussed in Section 4. In the EBSD image taken at the center of the sample annealed at 740 K, there are poorly indexed regions where the color code indicating the orientation changes from pixel to pixel. These regions were excluded in the determination of the grain size. Uncertain indexing may occur when the step size of the EBSD scan is not small enough for revealing the details of a fine microstructure. A part of the image in Figure 12b is magnified in Figure 12c without changing the scan step size (2 µm). The same area is shown in Figure 12d with a smaller step size of 0.5 µm, and this EBSD image unfolds a very fine lamellar microstructure. Indeed, the thickness of the almost vertical long lamellas is about 4 µm, and there are also transversal thinner lamellas with a thickness of ~1 µm. At 890 and 1000 K of annealing, the grain sizes of the disk centers were 15 ± 1 and 22 ± 4 µm, respectively, as determined from Figure 12e,f.

**Figure 13:**
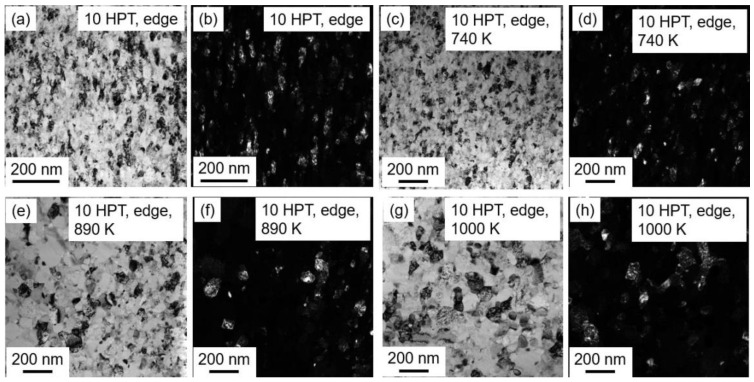
TEM BF (**a**,**c**,**e**,**g**) and DF (**b**,**d**,**f**,**h**) micrographs on the nanostructure obtained for the highest studied strain, i.e., for the edge of the disk processed by 10 turns of HPT. (**a**,**b**) immediately after HPT, (**c**,**d**) at 740 K, (**e**,**f**) at 890 K and (**g**,**h**) at 1000 K.

The evolution of grain size during annealing was studied by TEM for the highest strain, i.e., for the disk edge after HPT processing for 10 turns. Examples of the TEM images can be seen in Figure 13, where for each state of the HPT-processed and annealed samples a pair of BF and DF micrographs are shown. Immediately after HPT, the grain size was about 33 ± 1 nm, which remained practically unchanged after annealing at 740 K (38 ± 2 nm). The heat treatment at 890 K resulted in an increase in the grain size to 81 ± 10 nm, while further rise of the temperature to 1000 K caused the grain size to remain unchanged (87 ± 9 nm).

**Figure 14:**
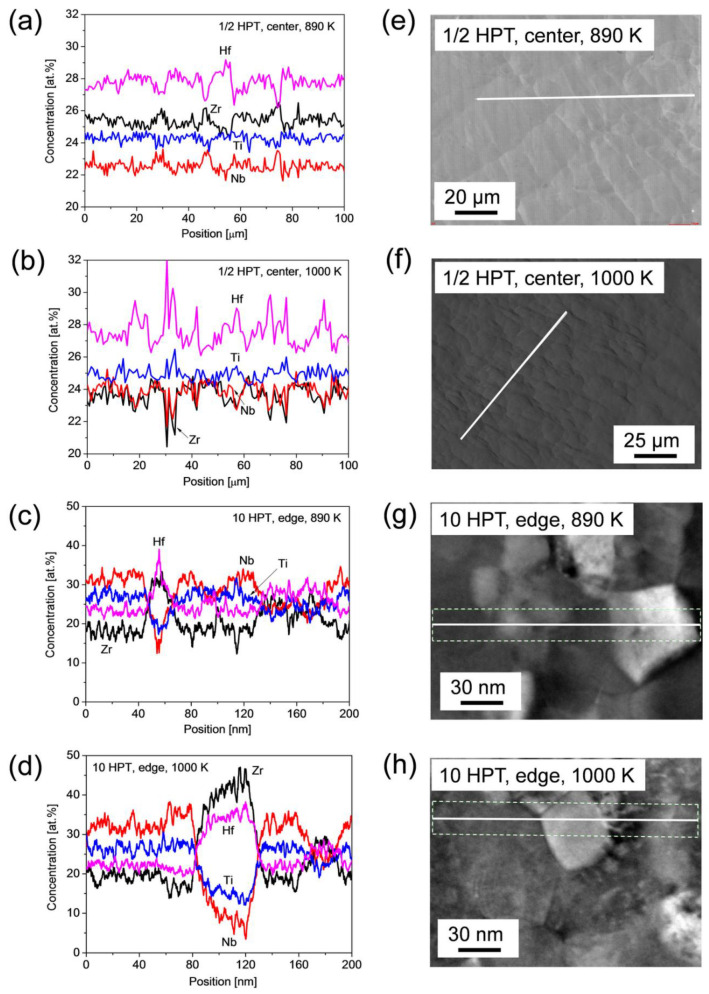
Elemental distributions of the four constituents measured along a line using EDS after the heat treatments at 890 and 1000 K for (**a**,**b**) the center of the disk processed for ½ turn (SEM-EDS), and (**c**,**d**) the edge of the sample deformed by HPT for 10 turns (TEM-EDS). The EDS distributions presented in (**a**–**d**) were obtained along the white straight lines shown in (**e**–**h**), respectively. For TEM-EDS, in each point the chemical composition was obtained by averaging the values perpendicular to the straight line inside the white dashed rectangle shown in (**g**,**h**).

The splitting of the XRD peaks of the HfNbTiZr phase into two profiles during annealing suggests a decomposition of the single bcc phase into bccL and bccH phases, which may be associated with the development of chemical heterogeneities. Therefore, the elemental distribution of the four constituents was measured along a line using EDS after the heat treatments. Significant chemical inhomogeneities were found only for 890 and 1000 K; therefore, the elemental distributions for these two temperatures are shown in Figure 14. Although the bcc-phase decomposition started as early as at 740 K, considerable chemical heterogeneities were not observed by the EDS line scan. This effect can be explained by the fourfold lower fraction of the bccL phase compared to the bccH phase at 740 K, where the probe line may overlook the former phase. It is suggested from Figure 14a,b that the chemical composition is not strictly equimolar. From numerous SEM-EDS analyses, the average atomic concentrations of Hf, Nb, Ti and Zr were 28 ± 2, 23 ± 2, 24 ± 2 and 25 ± 2%, respectively. In addition to the statistical error, an uncertainty of the constituent concentrations can emerge from the optional selection of lines K or L in the EDS spectrum. The absolute value of this uncertainty is about ±3 at.% in the average element concentrations. In the present EDS studies performed by SEM and TEM, line K was used for Ti while for the other three elements line L was evaluated for the determination of the atomic concentrations. Nevertheless, the detection of the chemical heterogeneities of the spatial elemental distribution was not influenced by the selection of the EDS spectrum lines.

Figure 14a,b show that for the sample that received the lowest strain (i.e., at the disk center after a half HPT turn) the heat treatment at 890 and 1000 K caused only slight chemical inhomogeneities. Namely, the local change in the atomic concentrations was less than 3%. For Ti, the concentration fluctuations were negligible, while the variation in the Hf content was complementary to the change in elements Nb and Zr. On the other hand, for the highest strain (i.e., at the edge of the disk processed by 10 turns) after annealing at 890 and 1000 K, the element pair Zr-Hf varied in an opposite way to the pair Nb-Ti. In addition, the magnitude of the concentration change was much larger for the sample with higher strain, such as 10–20 at.% differences at some locations as revealed in Figure 14c,d. The length scale of the concentration variation was about 10 µm for the center of the disk after a half turn. On the other hand, for the highest strain, the length scale of the chemical inhomogeneities was only 20–40 nm, i.e., three orders of magnitude smaller than that for the lowest studied strain.

### 3.5. Evolution of Hardness Attributed to Heat Treatment after HPT Straining

**Figure 15:**
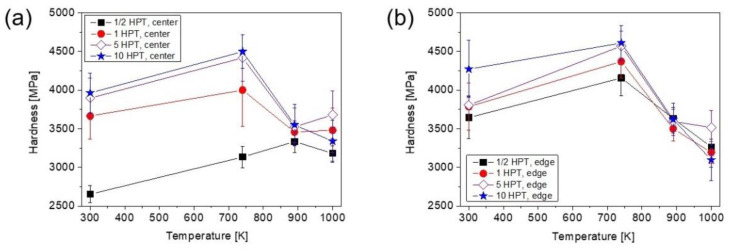
The hardness vs. the annealing temperature for different numbers of turns in the center (**a**) and the edge (**b**) parts of the disks processed by HPT.

**Figure 16:**
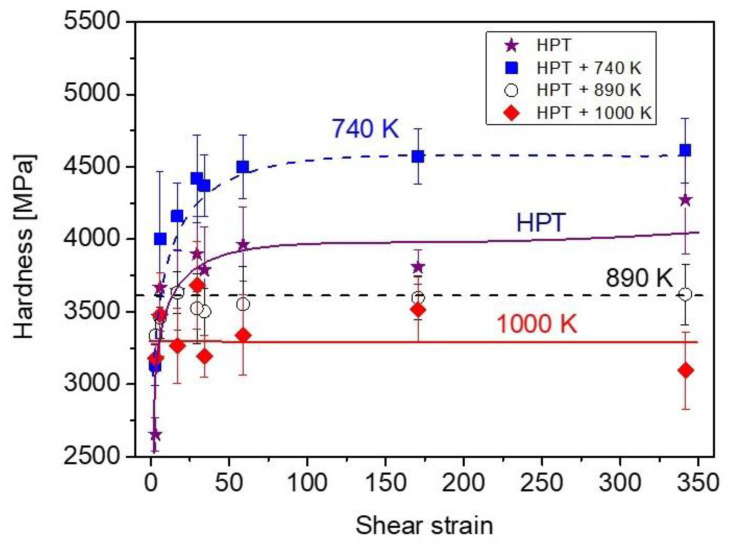
The hardness vs. the shear strain for the HPT-processed and the annealed states. The dashed and solid curves are just a guide for the eyes.

Figure 15a,b show the evolution of hardness as a function of the annealing temperature for the different sample locations, centers and edges, after different numbers of HPT turns. For a half turn, the hardness considerably increased after heating up to 740 K at both the disk center and edge after HPT. Although annealing at 740 K led to a hardness enhancement for all other numbers of turns at both the center and edge regions, for some samples this change was not significant if the errors of the hardness values are considered. Between 740 and 890 K, the hardness decreased, except for the center of the disk processed for a half turn. For all specimens, no or negligible variation in the hardness was detected when the temperature increased from 890 to 1000 K. Changes in hardness as a function of imposed shear strain is plotted in Figure 16 for the HPT-processed and the annealed samples. The samples immediately after HPT as well as the processed samples after being annealed at 740 K showed increased hardness with increasing strain, and was then saturated at shear strains of 20–30. On the other hand, at 890 and 1000 K, there was no significant variations in hardness vs. shear strain. Figure 16 also reveals that annealing at 740 K caused hardening above a strain of 20–30, while the further increase in the temperature to 890 K resulted in a softening, and between 890 and 1000 K only a negligible change occurred.

## 4. Discussion

### 4.1. Influence of SPD-Straining on the Annealing-Induced Changes in the Phase Composition and Microstructure

In this study, the thermal stability of an HfNbTiZr MPEA processed by HPT for a broad range of shear strain between ~3 and ~340 was investigated. For all studied strains, an initial single-phase bcc structure decomposed into two separate bcc phases having lower and higher values of lattice constant, which is more dominant with increasing annealing temperature. In the sample with high strains, an additional hcp phase was also developed during annealing. The decomposition of the single bcc phase is consistent with former Calphad thermodynamic calculation performed on a similar MPEA composition (HfNbTaTiZr) [31]. The earlier study predicted a two-phase microstructure with a Zr/Hf/Ti-rich hcp main phase with the coexistence of a Nb/Ta-rich bcc structure below a temperature of about 970 K. Above 970 K, the hcp phase was gradually substituted with a Ti/Zr/Hf-rich bcc structure in the HfNbTaTiZr MPEA. Similar to the HfNbTaTiZr HEA, the presently studied single-phase bcc HfNbTiZr alloy immediately after HPT was most probably far from equilibrium. In addition, the HPT samples contained a high number of lattice defects, such as dislocations and grain boundaries. Therefore, both phase transformation and recovery/recrystallization were expected to occur when the atomic mobility increased due to annealing. It should be noted, however, that the heat treatments in the present study were relatively short, since the samples were warmed up to the desired temperature at a heating rate of 40 K/min and then immediately quenched to RT. Therefore, the phase composition after annealing may be far from equilibrium even at the highest applied temperature (1000 K). Indeed, for low strains (e.g., for the center of the disk processed by a half turn) an hcp phase was not formed, and only the single bcc phase decomposed into bccL and bccH structures.

At the lowest selected temperature (740 K), the fraction of the bccL phase was low (20–30%). On the other hand, at 890 K the fractions of the two bcc phases became comparable; therefore, this decomposition is also reflected in the spatial distribution of the chemical composition. Indeed, Figure 14a reveals compositional fluctuations, where the local increase in Hf content was accompanied by a reduction in the concentrations of Zr and Nb. The Ti content fluctuation is not significant. The concentration variation in the two largest elements of Hf and Zr are complementary and their sizes are similar, while Nb is small; thus, the regions with elevated Hf concentrations have an increased lattice constant, i.e., they most probably correspond to a bccL phase. At 1000 K, the concentration fluctuations became higher and a significant variation was observed for Ti. Namely, Ti changed in a similar way as Hf. Since Ti is a small element, similar to Nb, the complementary variation in the element pairs Hf-Ti and Zr-Nb should reduce the lattice constant differences in the material. This is in accordance with the change in the XRD peak shape for the center of the disk processed for a half turn when the annealing temperature increased from 890 to 1000 K (see Figure 6a). Indeed, the double peak at 890 K became almost a single reflection at 1000 K, although a weak peak for bccL phase still existed.

For high strains (e.g., at the disk edge after HPT for 10 turns), an hcp phase was also formed and its quantity increased with increasing annealing temperature. For both 890 and 1000 K, the hcp phase fraction reached its saturation value at a shear strain of about 20–30. Grain boundaries are usually preferred sites for the nucleation of new phases; therefore, the introduced nanocrystalline microstructure at high HPT strains promoted the thermodynamically more stable hcp phase. This is the reason why this phase appeared only at sample conditions involving high shear strains. A former study [5] on the HfNbTiZr alloy revealed that the hcp phase is enriched by Zr and Hf, which is consistent with the Calphad estimation [31]. Simultaneously, the Ti and Nb content decreased in the hcp phase. The low amount of Nb in the hcp phase is reasonable, since Nb plays the role of bcc stabilizer (the other three elements form hcp phase in pure form). Accordingly, in the elemental distribution as shown in Figure 14c,d the concentrations of element pairs of Hf-Zr and Nb-Ti change in a complementary way. This behavior is significantly different from that observed for low strains, where the complementary element pairs were Hf-Ti and Zr-Nb (see Figure 14a,b). In the case of low strains, the grain size was large (about 20–30 µm), i.e., the number of grain boundaries was relatively low for all testing temperatures. Therefore, nucleation of the Hf/Zr-rich hcp phase is difficult during the applied short heat treatments, but instead only a chemical decomposition occurred, yielding two bcc phases with different lattice constants. When this decomposition took place, the lattice distortion at the boundaries separating the two bcc phases was moderated if the lattice constants of the bccL and bccH phases were not very different. Accordingly, the increase in the concentration of a large element (e.g., Zr) was accompanied by the enhancement of the concentration of a small element (e.g., Nb) in the same region. With increasing the temperature from 890 to 1000 K, the lattice constants of the two bcc phases became closer, as suggested by the change in the shape of the XRD peak in Figure 6.

The high number of grain boundaries in the nanocrystalline samples processed for high HPT strains facilitated not only the development of the hcp phase but also the recrystallization of the bcc phase. Indeed, at the edge of the disk deformed for 10 turns, the dislocation density in the bccL phase at 1000 K was below the detection limit of XLPA (~0.1 × 10^14^ m^−2^), which suggests the occurrence of recrystallization. On the other hand, significant dislocation density was detected at the center of the sample processed for a half turn.

### 4.2. Effect of the HPT Strain on the Hardness Change through Heat Treatment

Annealing at 740 K resulted in an 8–20% increase in hardness as shown in Figure 15. For some samples, this hardness change is uncertain due to the experimental error (about 5%); however, such a hardness increase was observed for all studied samples with various strains, suggesting that the hardening at 740 K is real. Former studies [32,33,34,35,36,37,38,39,40,41,42,43] have also revealed annealing-induced hardening of SPD-processed metallic materials if the heat treatment is short (not longer than 1 h) and the temperature is between 0.3 and 0.4 × *T_m_*, where *T_m_* is the melting point. Since the melting temperature of the HfNbTiZr MPEA is about 2058 K, the expected temperature range of anneal hardening is 620–820 K, that is, in accordance with the observed hardness increase at 740 K in the present study. There may be different reasons for this phenomenon, such as (i) the annihilation of mobile dislocations; (ii) the clustering of the remaining dislocations into boundaries, thereby refining the microstructure; (iii) the relaxation of SPD-processed non-equilibrium grain boundaries and (iv) the segregation of solute atoms from the solid solution phase at grain boundaries [43]. The latter two effects contribute to a more difficult dislocation emission from grain boundaries, thereby leading to hardening. In the present case, at low strains only the effects of (i) and (ii) may be the origins of hardening at 740 K, since the other two phenomena cannot play a significant role due to the coarse-grained microstructure. Former studies have shown that the strengthening effect of a clustered dislocation arrangement is higher than that for a uniformly distributed dislocation population [44,45]. For high strains (e.g., at the edge of the disk processed for 10 turns), due to the nanocrystalline microstructure, the grain boundary relaxation most probably plays a significant role for anneal hardening.

When the temperature of heat treatment changed from 740 to 890 K, additional significant hardening was not observed at the center of the disk deformed for a half turn in accordance with the unchanged grain size. On the other hand, considerable softening occurred for the other samples with increasing temperature from 740 to 890 K. The detailed microstructure analysis at the disk edge after 10 HPT turns revealed a coarsening of the nanostructure, since the grain size increased from ~38 to ~81 nm. This effect and the decrease in the dislocation density inside the grains can cause the observed softening. Between 890 and 1000 K, only a slight reduction or no change in hardness was observed for both low and high strains. This can be explained by the unchanged or slightly increased grain size at 1000 K as compared to the microstructure at 890 K.

## 5. Conclusions

Experiments were conducted to study the effect of SPD straining on the evolution of microstructure, phase composition and hardness of an HfNbTiZr MPEA processed by HPT. The following conclusions were obtained in this study:

1. With increasing shear strain by HPT, the dislocation density and crystallite size increased and decreased, respectively, which then are saturated at a shear strain of ~30 with values of about 15 nm and 220 × 10^14^ m^−2^, respectively. XRD synchrotron experiments suggested an in-depth homogeneity of the microstructure in the HPT-processed disks for both low and high strains. When the shear strain increased from ~3 to ~340, the grain size decreased from about 20 µm to 30 nm.
2. A single bcc phase in the HfNbTiZr MPEA decomposed into two bcc phases even after the lowest annealing temperature of 740 K. Above 890 K, an hcp phase also appeared at the sample location having high strains. The hcp fraction increased with increasing strain at both 890 and 1000 K, and saturated at a shear strain of about 20 with values of 5 and 13%, respectively.
3. Significant chemical heterogeneities were observed in the samples with both low and high strains followed by annealing at 890 and 1000 K. These inhomogeneities are most probably related to the phase decomposition. With increasing SPD strain, the scale of the heterogeneities varied in a similar way as the grain size. Namely, it decreased from about 10 µm to 30 nm when the shear strain increased from ~3 to ~340.
4. Anneal-induced hardening was observed after heat treatment at 740 K. The maximum hardness was achieved at the edge of the disk processed by 10 turns of HPT (which corresponds to a shear strain of about 340) and annealed at 740 K (~4600 MPa). The relative hardening was in the range of 8–18% for the shear strains between ~3 and ~340. The anneal hardening can be attributed to the annihilation of mobile dislocations and their arrangement into boundaries. For low strain, the hardness remained practically unchanged between 740 and 1000 K. On the other hand, for high strains there was a reduction in hardness between 740 and 890 K due to the coarsening of the microstructure.

## Data Availability

The measured data of this study are available on request from the corresponding author.
